# European cancer research: from bench to bedside and to breakfast table

**DOI:** 10.3332/ecancer.2016.ed60

**Published:** 2016-11-02

**Authors:** Mursheda Begum, Elena Pallari, Grant Lewison

**Affiliations:** King’s College London, Department of Cancer Studies, London SE1 6RT, UK

**Keywords:** cancer research, impacts, funding, clinical guidelines, newspaper stories

## Abstract

We examined the outputs, sources of funding, and impact of European cancer research from 2002-13. Outputs were compared with the disease burden and individual countries’ wealth. Funding came from a huge number of sources, particularly private-non-profit ones in northern and western Europe. Impacts were determined from citations on cancer clinical guidelines and in European newspaper stories about cancer research.

We have recently completed a major mapping exercise for the European Commission on research outputs in cancer and four other non-communicable diseases (cardiovascular disease and stroke, diabetes, mental disorders and respiratory diseases). We identified papers in the Web of Science (WoS) in one of 185 specialist journals, and/or containing one or more of 323 title words or phrases, and with an address in Europe (the European Union 28, plus Iceland, Norway and Switzerland) from 2002-13. We examined their subject matter and their impacts (Begum & Lewison, 2014); these included academic citations, but of greater practical importance, the references on European clinical guidelines and the papers cited in stories in 30 European newspapers.

The file of cancer papers comprised just over 282,000 papers. We compared countries’ outputs with their wealth (gross domestic product), which were quite closely correlated. Iceland, Croatia and Slovenia performed particularly well, with outputs more than twice as high as the regression line would suggest, but Luxembourg and Latvia published less than half, and Cyprus and even France performed rather poorly on this criterion. Overall, some 11 % of European biomedical research was on cancer.

The burden of disease from cancer in Europe averaged 16% of Disability-Adjusted Life Years (DALYs) in 2002 and 19.5% in 2012 according to the WHO. So cancer was relatively under-researched in Europe according to this criterion, although world-wide it appears over-researched since the disease burden was only 5% in 2002 and 8% in 2012, and cancer research rose from 12% to 13% in these years. There is an imbalance between European burden from different cancer sites and research output, with the latter dominated by breast cancer (10%) whereas the burden is primarily from lung cancer (21% of the cancer total).

There is also an imbalance in the relative effort on the three main means of treatment. Chemotherapy and surgery each account for 11% of European cancer research, but radiotherapy only for 5%. Palliative care is also relatively neglected with 1.2% of the total, especially in central and eastern Europe, but also France and Germany, though it is quite strong in Norway, the other Scandinavian countries and the UK. Clearly there is a need for many countries to re-balance their cancer research portfolios to take more account of their disease burden from different cancers and the needs for treatment.

But this may be difficult because of the huge number of organisations that fund cancer research in Europe. Although there are large state agencies such as INSERM in France, the Deutsche ForschungsGesellschaft in Germany, the Consiglio Nazionale delle Ricerche in Italy and the Medical Research Council and the National Institute of Health Research in the UK, in western Europe their support takes second place to the numerous private-non-profit funders. These include collecting charities, some also very large such as Cancer Research UK and the German Cancer Society, but many of them tiny, and set up to support research on individual rare cancers, or to commemorate the untimely death of a much-loved family member. There are literally thousands of these, and of endowed foundations, including almost 250 in Denmark alone, most named for a successful entrepreneur and his wife. Co-ordination of all these funders would make herding cats seem relatively easy, and a first step would probably be for countries to create umbrella groups comparable to the Association of Medical Research Charities in the UK. These could mandate standards such as external peer review of proposals, oversee the publication of financial information and lists of projects, and lobby national governments for the easing of burdensome restrictions on research and improve the fiscal regime governing medical research charities. The existence of a vibrant private-non-profit sector with contestable research funding is likely to be one of the main reasons for the superior academic citation performance of cancer research in Switzerland, the Netherlands and the UK.

Cancer clinical guidelines have been proliferating in Europe during the 21st century, both nationally and regionally. For our study, we were assisted by our five continental European partners, volunteers from Hungary and Sweden, and King’s College graduate students with appropriate national backgrounds, to identify relevant guidelines from the web. We limited our collection of references to ones on guidelines for three common solid cancers: lung, breast and colorectal. In total, we collected 9978 cited papers from 71 different guidelines to form a guidelines evidence database; these papers could be compared with those in the WoS for cancer in the same years. The most striking findings were two. First, we noted that most European countries’ research was relatively over-cited compared to its percentage presence in the world, especially that from Belgium (x 4.3); Denmark and the Netherlands (x 3.2); and Ireland, Poland, Switzerland and the UK (x 2.7). Conversely, research from the major east Asian countries (China, Japan, South Korea and Taiwan) was relatively neglected despite most of it being published in English. And second, we saw that surgery and radiotherapy research were much more prominent among guideline citations than that on genetics, which is currently the most fashionable subject area for cancer research and the most cited academically.

Newspapers, despite falling print circulations, remain very influential and some, such as the UK’s *Daily Mail*, often feature news of medical research “breakthroughs” in banner headlines on their front page. Newspapers can (and do) influence politicians, their senior advisers and administrators, medical personnel, researchers and the general public. [We are increasingly encouraged to be healthcare consumers rather than mere patients.] Compared with social media, newspapers provide a relatively permanent record that can be searched *via* their individual archives or through full-text databases such as Factiva. Our team were set to work to search a total of 30 newspapers from 22 different countries, and decide which stories were relevant (because they cited identifiable research papers in the WoS) - many were not. They then entered salient details of the stories, including codes describing the subject matter, and of the cited papers to a standardised spreadsheet. This now has over 3200 stories about cancer research. We noted that the majority concerned cancer avoidance (epidemiology and genetics) rather than treatment, but it is still taking many years to get the standard health messages across: don’t smoke or don’t start; drink very moderately or not at all; eat good food, preferably a Mediterranean diet; and exercise regularly. Perhaps the messages have become dulled by repetition but they may slowly bring about more acceptance of national legislation designed to encourage healthy living. Our other finding was the relatively low output of stories on surgery and radiotherapy, so that the public and politicians will come to believe that the “war on cancer” depends primary on new and expensive drugs from big pharma, with a distorting effect on health spending.

What of the future? We are completing our analyses and adding to the flood of papers on medical bibliometrics, of course. But we also want to exploit our two databases of clinical guideline references and of papers cited in newspaper stories to enable the funders and performers of cancer research in Europe to evaluate their outputs for their real-world impact. We envisage the creation of a “club” project in which we would inform members, who would pay a graduated annual subscription, which of their published papers had been cited on one (or more) guidelines or in European newspapers. Some papers have been cited 11 times on our existing, necessarily incomplete, set of guidelines, and up to 13 times in our collection of European newspapers. These are the new “highly cited” papers which should attract approbation for their funders and researchers, and help to spread best practice in cancer research across Europe.
